# Indications, safety and aesthetic outcomes of autologous conversion after implant-based breast reconstruction failure: A systematic review and meta-analysis

**DOI:** 10.1016/j.jpra.2026.08.029

**Published:** 2026-08-25

**Authors:** Theodore Lahmar, François Thuau, Anoujat Kanlagna, Ugo Lancien, Pierre Perrot

**Affiliations:** a Department of Plastic and Reconstructive Surgery, University Hospital of Nantes, 1 place Alexis Ricordeau, 44000, Nantes, France; b RMeS: Regenerative Medicine and Skeleton research unit - Inserm, Nantes University, 44000, Nantes, France

**Keywords:** Autologous conversion, Implant-based breast reconstruction, Autologous breast reconstruction, Implant failure, Post-mastectomy radiotherapy

## Abstract

**Background:**

Implant-based breast reconstruction (IBBR) is widely used after mastectomy but is limited by implant-related morbidity and failure, particularly in irradiated patients. Autologous conversion (implant removal followed by flap reconstruction) is increasingly performed, yet indications, safety, and aesthetic outcomes remain inconsistently reported.

**Methods:**

A systematic review was conducted according to PRISMA 2020. PubMed, Scopus, and Cochrane CENTRAL were searched from inception to January 2026. Studies reporting implant removal followed by autologous reconstruction after mastectomy were included. Primary outcomes were (1) indications for conversion and (2) surgical safety, including major postoperative complications and total flap loss. Safety outcomes were pooled using random-effects meta-analysis of proportions; comparative meta-analysis versus primary autologous breast reconstruction, including immediate, delayed, or delayed-immediate reconstruction (P-ABR), was conducted when eligible data were available. Aesthetic outcomes were synthesized descriptively.

**Results:**

Nineteen studies (1994–2025) including 1304 patients and 1552 reconstructed breasts were included. Conversion was predominantly performed using free flaps (91.5%), most commonly abdominal-based reconstructions (72.8%). Most conversions were elective, primarily for capsular contracture and dissatisfaction; obligatory implant removal leading to conversions were mainly driven by infection and soft-tissue compromise. The pooled major complication rate was 10.8% (95% CI, [8.12–14.19]; I² = 52.9%) and pooled total flap loss was 2.00% (95% CI, [1.43–2.83]; I² = 0%). Comparative analysis showed no statistically significant difference in major complications between conversion and P-ABR (RR, 1.68; 95% CI, [0.70–4.01]; I² = 0%). Pre-post analyses or comparison with continued IBBR showed improved aesthetic outcomes after conversion.

**Conclusions:**

Autologous conversion after IBBR is most often performed electively for chronic implant-related morbidity or dissatisfaction and demonstrates low flap loss with acceptable complication rates.

## Introduction

Breast cancer remains the most commonly diagnosed malignancy among women worldwide,^1^^,^^2^ and mastectomy continues to play a central role in surgical management.^3^^,^^4^ In this context, implant-based breast reconstruction (IBBR) remains the most frequently performed reconstructive strategy in many healthcare systems, supported by shorter operative times, broad availability, and perceived lower early morbidity compared with autologous reconstruction.^5^, ^6^, ^7^

However, long-term outcomes following IBBR are heterogeneous and often limited by implant-related morbidity, particularly among patients exposed to post-mastectomy radiation therapy.^8^, ^9^, ^10^ Complications such as capsular contracture, infection, implant malposition, asymmetry, rupture, extrusion, and recurrent revisional surgery may ultimately result in reconstructive failure and contribute to chronic discomfort and aesthetic dissatisfaction.^11^, ^12^, ^13^, ^14^ Even with adjunctive soft-tissue coverage strategies, these risks are not fully mitigated, highlighting the structural limitations of prosthetic reconstruction in compromised tissue environments.^15^^,^^16^

Autologous breast reconstruction (ABR) using flap-based techniques provides a durable tissue-based alternative and has demonstrated favorable long-term stability and patient-reported outcomes,^17^, ^18^, ^19^, ^20^ albeit at the expense of increased operative complexity and donor-site morbidity.^21^^,^^22^ As implant-related complications and dissatisfaction accumulate over time, an increasing number of patients undergo implant removal followed by flap-based reconstruction, a process referred to as autologous conversion and also described in the literature as tertiary reconstruction or salvage autologous breast reconstruction.^23^^,^^24^

Despite its increasing clinical relevance, evidence specific to autologous conversion remains fragmented and inconsistently reported. In particular, the indications leading to implant removal and subsequent conversion, as well as the surgical safety of performing flap reconstruction in previously operated and altered tissue environments, remain incompletely characterized. These uncertainties limit patient counseling, shared decision-making, and perioperative risk stratification when managing implant failure or persistent dissatisfaction.

Therefore, this systematic review aimed to synthesize the available evidence to: (1) characterize the indications and motivations leading to implant removal and autologous conversion; (2) evaluate the safety of autologous conversion through pooled estimates of major complications and total flap loss, including comparative analyses versus primary (immediate, delayed, or delayed-immediate) autologous breast reconstruction (P-ABR) when available; and (3) assess aesthetic outcomes following autologous conversion.

## Methods

### Study design and search strategy

This systematic review and quantitative synthesis was conducted in accordance with the Preferred Reporting Items for Systematic Reviews and Meta-Analyses (PRISMA 2020) guidelines.^25^ The analytical framework was prespecified, and the protocol was prospectively registered in PROSPERO (CRD420261302397).

A comprehensive literature search was performed in PubMed (MEDLINE), Scopus, and the Cochrane Central Register of Controlled Trials, without date restrictions. The final search was conducted in January 2026. Search terms combined controlled vocabulary and free-text terms related to breast reconstruction and implant failure, with database-specific adaptations, including: (Reconstruction) AND (Breast) AND ((Implant) OR (failure) OR (removal)) AND ((conversion) OR (secondary) OR (tertiary) OR (Salvage)). Reference lists of included studies and relevant review articles were manually screened for additional eligible records.

### Eligibility criteria and study selection

All study designs were eligible (single-arm cohort studies, comparative observational studies, interventional studies, and randomized controlled trials). Studies were included if they reported outcomes among patients undergoing implant removal followed by autologous reconstruction using a free or pedicled flap after prior IBBR following mastectomy for therapeutic or prophylactic indications. Studies were required to report data on at least one prespecified outcome domain.

Studies were excluded if they: (1) focused on planned delayed-immediate or expander-to-flap strategies; (2) included mixed flap-implant techniques within the conversion cohort without separable data; (3) involved non-mastectomy indications; (4) were non-original research (reviews, editorials); (5) included overlapping cohorts (in which case the most comprehensive or most recent dataset was retained); (6) restricted the conversion cohort to a single exclusive indication for implant removal; or (7) were small case series (<10 patients). Only studies published in English or French were considered.

After duplicate removal, titles and abstracts were independently screened by two reviewers (TL, PP), followed by full-text assessment. Disagreements were resolved by consensus or third-party adjudication (UL).

### Outcomes

The primary outcomes were: (1) indications and motivations leading to implant removal and autologous conversion; and (2) surgical safety outcomes following autologous conversion, including overall postoperative complications, major postoperative complications and total flap loss.

Secondary outcomes included the clinical profile and oncologic exposure of patients undergoing conversion, and aesthetic outcomes following conversion. Aesthetic outcomes were broadly defined as any study-reported evaluation of reconstructed breast appearance, aesthetic result, or patient-perceived satisfaction with the appearance of the reconstructed breast. Eligible measures included aesthetic assessments, such as clinician- or observer-rated evaluations of reconstructed breast aesthetics, and patient-reported assessments, including validated patient-reported outcome measures such as the BREAST-Q© Satisfaction with Breasts domain, as well as non-validated questionnaires, provided they specifically addressed breast appearance or aesthetic satisfaction.

### Definitions

Indications for autologous conversion were classified a priori into three mutually exclusive categories at the patient level whenever possible: (1) Elective surgery was defined as implant removal leading to autologous reconstruction performed for chronic implant-related morbidity, including capsular contracture, aesthetic or general dissatisfaction, implant-related disorders (e.g., malposition, animation deformity), or functional symptoms such as pain or discomfort; (2) Obligatory surgery was defined as implant removal leading to conversion performed following an acute implant-related complication requiring explantation, including infection, soft-tissue compromise (exposure, extrusion, or skin necrosis), or implant rupture; (3) Unknown, when the primary indication could not be reliably determined.

Surgical safety outcomes were analyzed per reconstructed breast: (1) Major postoperative complications were defined a priori as complications requiring return to the operating room, partial or total flap loss. Complications that could not be reliably mapped to a return-to-operating-room event were excluded from the major complication analysis; (2) Total flap loss was defined as complete loss of the transferred flap, irrespective of the timing; (3) Overall postoperative complications were defined as any complication reported by the study authors as related to the reconstructive procedure.

### Data extraction

Data were extracted independently by two reviewers (TL, PP) using a standardized spreadsheet (Microsoft Excel, Microsoft Corp., Redmond, WA, USA). Extracted variables included study characteristics, demographics, oncologic exposures, indications for conversion, reconstructive techniques, follow-up duration, and postoperative outcomes. Corresponding authors were contacted when clarification or additional data were required.

### Quality assessment

Risk of bias was independently assessed by two reviewers using the Newcastle-Ottawa Scale (NOS) for cohort studies. The NOS evaluates participant selection, comparability of study groups, and outcome ascertainment. Disagreements were resolved through discussion. Risk of bias assessments informed interpretation of findings but were not used as exclusion criteria.

### Data synthesis and statistical analysis

Baseline clinical and oncologic characteristics were summarized descriptively. Because indications were frequently non-mutually exclusive and multiple reasons could be recorded per patient, formal meta-analysis of proportions was not performed. Indication and motivations were synthesized using complementary descriptive approaches, including patient-level analyses restricted to studies reporting a single primary indication per patient using mutually exclusive categories classified a priori as elective, obligatory, or unknown, and event-level analyses in studies allowing multiple indications per patient, summarized separately within elective and obligatory categories using total reported events as the denominator. An exploratory descriptive analysis examined patterns in primary indications according to study-level radiotherapy burden, dichotomized as <50% versus ≥50%.

Safety outcomes were evaluated using single-arm random-effects meta-analyses of proportions to estimate pooled event rates for major complications and total flap loss per reconstructed breast. Proportions were pooled on the logit scale using restricted maximum likelihood estimation, with Hartung-Knapp adjustment for confidence intervals and back-transformation to the proportion scale. Study-level confidence intervals were calculated using the Clopper-Pearson method. Continuity correction was applied when zero-event studies were present. Between-study heterogeneity was assessed using I², τ², and the Cochran Q test, and prediction intervals were reported. For studies directly comparing conversion to P-ABR, comparative meta-analyses were performed using random-effects Mantel-Haenszel models and reported as risk ratios with 95% confidence intervals for major and overall postoperative complications.

Aesthetic outcomes were synthesized descriptively due to heterogeneity in assessment tools, follow-up duration, and reporting formats. No quantitative pooling was performed for these endpoints.

Single-arm analyses were performed in R (meta package; R Foundation for Statistical Computing), and comparative meta-analyses were performed using Review Manager (RevMan 5.4).

## Results

### Study selection and characteristics

**Fig. 1:**
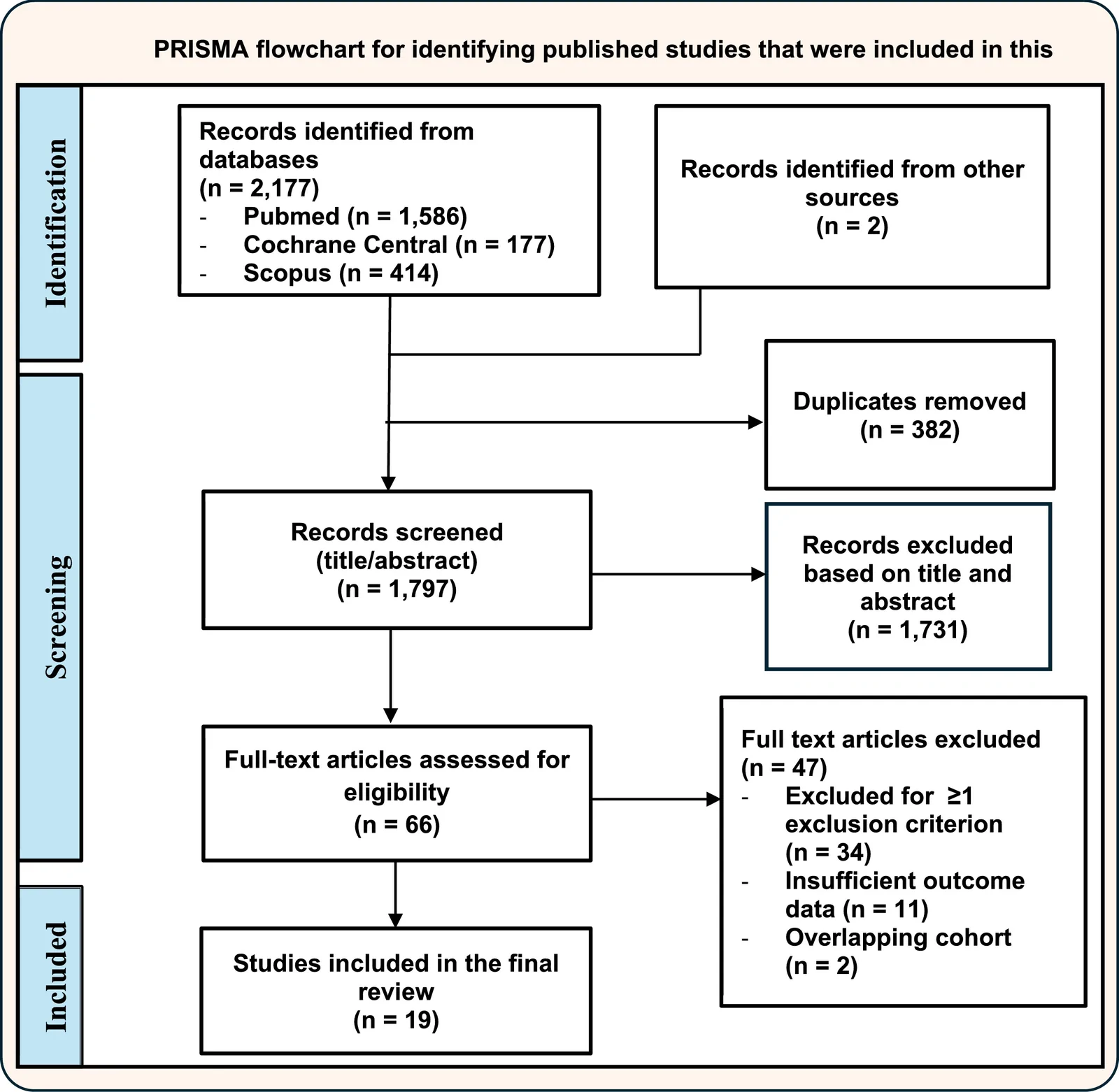
PRISMA flowchart for identifying published studies that were included in this review.

A total of 2177 records were identified through database searching using the predefined search strategy, including PubMed (n = 1586), Scopus (n = 414), and the Cochrane Central Register (n = 177). Two additional articles were identified through manual screening of reference lists from relevant studies. After removal of 382 duplicate records, 1797 titles and abstracts were screened, and 66 full-text articles were assessed for eligibility. Nineteen studies published between 1994 and 2025 were included in the review (Fig. 1).

Across 19 included studies, 18 reported indications and motivations for implant removal leading to autologous conversion and 19 contributed to safety analyses. Overall, 1304 patients underwent autologous conversion after IBBR. Across 1552 reconstructed breasts, conversion was predominantly performed using free flaps (n = 1421; 91.5%), with a marked predominance of abdominal-based free tissue transfer (n = 1130; 72.8%). The DIEP flap represented >55% of all reported reconstructions, followed by the free TRAM flap (13%). In contrast, pedicled flaps accounted for a minority of procedures (n = 90; 5.8%), and flap type was not reported in 41 reconstructions (2.6%). Most studies were retrospective cohorts, with fewer prospective single-arm series and comparative designs including either primary autologous or implant-based control groups (Table 1). With respect to indications and motivations for conversion, six studies reported a single primary indication per patient using mutually exclusive categories, whereas twelve studies allowed multiple indications per patient and were therefore analyzed at the event level. Regarding surgical safety, all included studies contributed to the single-arm-meta-analyses of proportions, while only five comparative studies provided data suitable for inclusion in the meta-analysis comparing autologous conversion with P-ABR.

**Table 1:** Summary of reported studies.

**Authors**	**Year**	**Country**	**Study** **Design**	**Data Collection** **(Years)**	**Overall Patients** **n**	**Type of Reconstruction** **(%)**	**Number of reconstructed breasts** **n**	**Indications and Motivations**	**Safety Analysis**
**Feng et al.**^56^	1994	USA	Retrospective single-arm cohort	1988–1993	33	Free Flap (83%) Pedicled Flap (17%)	32 fTRAM; 11 SGAP; 9 LD	Event-level	✓
**Visser et al.**^57^	2010	Canada	Retrospective single-arm cohort	2001–2007	42	Free Flap (100%)	47 DIEP; 4 TMG; 10 fTRAM	Event-level	✓
**Levine et al.**^24^	2011	USA	Prospective single-arm cohort	1998–2008	191	Free Flap (100%)	164 DIEP; 5 TMG; 30 SIEAP; 50 SGAP; 35 IGAP	Event-level	✓
**Hamdi et al.**^58^	2011	Belgium	Retrospective single-arm cohort	2002–2009	54	Free Flap (100%)	57 DIEP; 4 TMG; 9 SIEAP	Event-level	✓
**Rabey et al.**^23^	2013	UK	Retrospective single arm cohort	2000–2012	14	Free Flap (100%)	9 DIEP; 5 fTRAM	Event-level	✓
**Tadiparthi et al.**^59^	2013	UK	Retrospective comparative cohort (Conversion vs IBBR)	2000–2008	Conversion: 99	Free Flap (95%) Pedicled Flap (5%)	8 DIEP; 10 fTRAM; 1 LD	Patient-level	✓
					IBBR: 21				
**Roostaeian et al.**^60^	2016	USA	Retrospective comparative cohort (Conversion vs P-ABR)	2005–2014	Conversion: 89	Free Flap (100%)	72 DIEP; 33 fTRAM; 9 SIAEP; 1 LAP; 6 SGAP; 1 SCIP	Event-level	✓ (comparative meta-analysis)
					P-ABR: 89				
**Zhao et al.**^54^	2018	USA	Multicentre Retrospective comparative cohort (Elective vs Obligatory)	2006–2016	Elective: 68	Free Flap (97%) NR (3%)	90 DIEP; 21 fTRAM; 1 SIEAP; 3 NR	Event-level	✓
					Obligatory: 47				
**Coriddi et al.**^21^	2019	USA	Prospective single arm cohort	1997–2017	137	Free Flap (93%) Pedicled Flap (7%)	92 DIEP; 4 DUG; 79 fTRAM; 13 pTRAM; 4 SGAP	Patient-level	✓
**Holmes et al.**^61^	2019	UK	Retrospective single-arm cohort	1998–2016	151	Free Flap (100%)	151 DIEP	Patient-level	✓
**Cowen et al.**^62^	2020	France	Retrospective comparative cohort (Conversion vs IBBR)	2010–2018	Conversion: 13	Free Flap (54%) Pedicled Flap (46%)	5 DIEP; 6 LD; 2 TMG	Event-level	✓
					IBBR: 34				
**Shammas et al.**^37^	2022	USA	Retrospective comparative cohort (Conversion vs P-ABR)	2015–2019	Conversion:23	Free Flap (100%)	35 DIEP	Event-level	✓ (comparative meta-analysis)
					P-ABR: 108				
**Bojanic et al.**^63^	2023	UK	Retrospective comparative study	2005–2019	Conversion: 35	Free Flap (100%)	26 DIEP; 4 fTRAM; 5 SIEAP	Event-level	✓ (comparative meta-analysis)
					IBBR: 35				
**De Kerchkove et al.**^64^	2023	Japan	Retrospective single-arm cohort	1998–2019	Conversion: 23	Free Flap (100%)	7 DIEP; 16 fTRAM	Patient-level	✓
					IBBR: 819				
**Von Glinski et al.**^14^	2023	Germany	Retrospective comparative cohort (Conversion vs P-ABR)	2014–2020	Conversion: 23	Free Flap (100%)	30 DIEP; 3 fTRAM	Patient-level	✓ (comparative meta-analysis)
					P-ABR: 47				
**Couto-Gonzalez et al.**^65^	2024	Spain	Prospective single-arm cohort	2014–2020	56	Pedicled Flap (100%)	61 LD	Event-level	✓
**Plotsker et al.**^66^	2024	USA	Retrospective comparative cohort (Conversion vs IBBR)	2018–2021	Conversion: 33	NR	NR	-	✓
					IBBR: 307				
**Kim et al.**^67^	2025	USA	Retrospective comparative cohort (Conversion vs P-ABR)	2012–2023	Conversion: 119	Free Flap (96%) NR (4%)	68 DIEP; 27 PAP; 19 LAP; 5 NR	Event-level	✓ (comparative meta-analysis)
					P-ABR: 1329				
**Bigdeli et al.**^38^	2025	Germany	Retrospective comparative cohort (Elective vs Obligatory)	2015–2023	Elective: 101	Free Flap (100%)	66 DIEP or fTRAM; 54 TMG	Event-level	✓
					Obligatory: 19				

IBBR: Implant-based breast reconstruction; P-ABR: Primary (immediate or delayed) autologous breast reconstruction; DIEP: deep inferior epigastric perforator flap; pTRAM: pedicled transverse rectus abdominis muscle flap; fTRAM: free transverse rectus abdominis muscle flap; SIEAP: superficial epigastric inferior artery perforator flap; TMG: transverse myocutaneous gracilis flap; PAP: profunda artery perforator flap; LAP: lumbar artery perforator flap; SCIP: superficial circumflex iliac perforator; DUG: diagonal upper gracilis flap; LD: latissimus dorsi flap; NR: not reported.

### Quality assessment

Quality was assessed using the NOS (Table 2). For single-arm cohorts, no points were attributed for the items “selection of the non-exposed cohort” and “comparability of cohorts”, resulting in an effective maximum score of 6 rather than 9; within this framework, most single-cohorts studies achieved scores of 5 to 6. Comparative cohort studies generally demonstrated higher methodological quality, with total scores ranging from 7 to 9.

**Table 2:** Newcastle-Ottawa quality assessment form for cohort studies.

Study	Selection				Comparability	Outcome			Total score
	Representativeness of the Exposed Cohort	Selection of the Non-Exposed Cohort	Ascertainment of Exposure	Outcome of interest not present at start of study	Comparability of cohorts on the basis of the design or analysis	Assessment of outcome*	Follow-up Long Enough	Adequacy of Follow-up	Maximum: 9
**Single-arm cohort**									
Feng et al. 1994^56^	★	NA	★	★	NA	★	★	★	**6**
Visser et al. 2010^57^	★	NA	★	★	NA	★	★	★	**6**
Levine et al. 2011^24^	★	NA	★	★	NA	★	★	-	**5**
Hamdi et al. 2011^58^	★	NA	★	★	NA	★	★	**-**	**5**
Rabey et al. 2013^23^	★	NA	★	★	NA	★	★	★	**6**
Coriddi et al. 2019^21^	★	NA	★	★	NA	★	★	★	**6**
Holmes et al. 2019^61^	★	NA	★	★	NA	★	★	★	**6**
De Kerchkove et al. 2023^64^	★	NA	★	★	NA	★	★	★	**6**
Couto-Gonzalez et al. 2024^65^	★	NA	★	★	NA	★	★	**-**	**5**
**Comparative cohorts**									
Tadiparthi et al. 2013^59^	★	★	★	★	★	★	★	★	**8**
Roostaeian et al. 2016^60^	★	★	★	★	★★	★	★	★	**9**
Zhao et al. 2018^54^	★	★	★	★	★	★	★	-	**7**
Cowen et al. 2020^62^	★	★	★	★	★	★	★	-	**8**
Shammas et al. 2022^37^	★	★	★	★	★	★	★	**-**	**7**
Bojanic et al. 2023^63^	★	★	★	★	★	★	★	★	**8**
Von Glinski et al. 2023^14^	★	★	★	★	★	★	★	★	**8**
Plotsker et al. 2024^66^	★	★	★	★	★	★	★	★	**8**
Kim et al. 2025^67^	★	★	★	★	★	★	-	**-**	**6**
Bigdeli et al. 2025^38^	★	★	★	★	★	★	★	★	**8**

### Clinical profile and oncologic characteristics

Baseline clinical characteristics and oncologic treatment exposures were reported inconsistently across studies (Table 3). Among studies reporting age and body mass index, the study-level summary mean age was 50.15 years (SD 8.88; 18 studies; n = 1278), and the study-level summary mean BMI was 26.67 kg/m² (SD 4.81; 13 studies; n = 963). Reporting-dependent proportions were 18.9% for hypertension and/or diabetes (14 studies; 179/948), 7.8% for current smoking (13 studies; 58/744), and 20.9% for former smoking (7 studies; 126/604). Radiotherapy and chemotherapy were reported in 16 and 12 studies, respectively, and were present in 58.0% (608/1049), and 58.4% (418/716) of patients with available data; the study-level radiotherapy burden had a median of 63.1% (IQR 41.1–80.2). Hormone therapy was reported in four studies and was present in 27.8% (77/277). Prior revision surgery before conversion was reported in nine studies and occurred in 32.2% (238/740). Time to conversion was reported in nine studies, with a study-level summary mean of 5.1 years (SD 3.58; n = 491).

**Table 3:** Study-level clinical profile and oncologic treatment exposure of patients undergoing autologous conversion.

Variable (Clinical profile)	Contributing studies / total (n = 19)	Patients with data (n)	Summary
Age (years)	18 / 19	1278	50.15 ± 8.88 ‡
BMI (kg/m²)	13 / 19	963	26.67 ± 4.81 ‡
Comorbidities*	14 / 19	179 / 948	18.9% (11.4–26.0) †
Current smoker	13 / 19	58 / 744	7.8% (1.5–15.0) †
Former smoker	7 / 19	126 / 604	20.9% (16.5–28.0) †
Radiation therapy §	16 / 19	608 / 1049	58.0% (41.1–80.2) †
Chemotherapy	12 / 19	418 / 716	58.4% (49.2–67.9) †
Hormone therapy	4 / 19	77 / 277	27.8% (8.1–40.2) †
Prior revision surgeries (≥1)	9/19	238 / 740	32.2% (21.5–45.0) †
Time to conversion (years)	9/19	491	5.1 ± 3.58 ‡

⁎ Comorbidities were defined as hypertension and/or diabetes; § Radiation therapy included pre- or post-mastectomy radiation therapy; ‡Mean per study ± standard deviation; †Median per study (interquartile range).

### Indications and motivations leading to autologous conversion

**Fig. 2:**
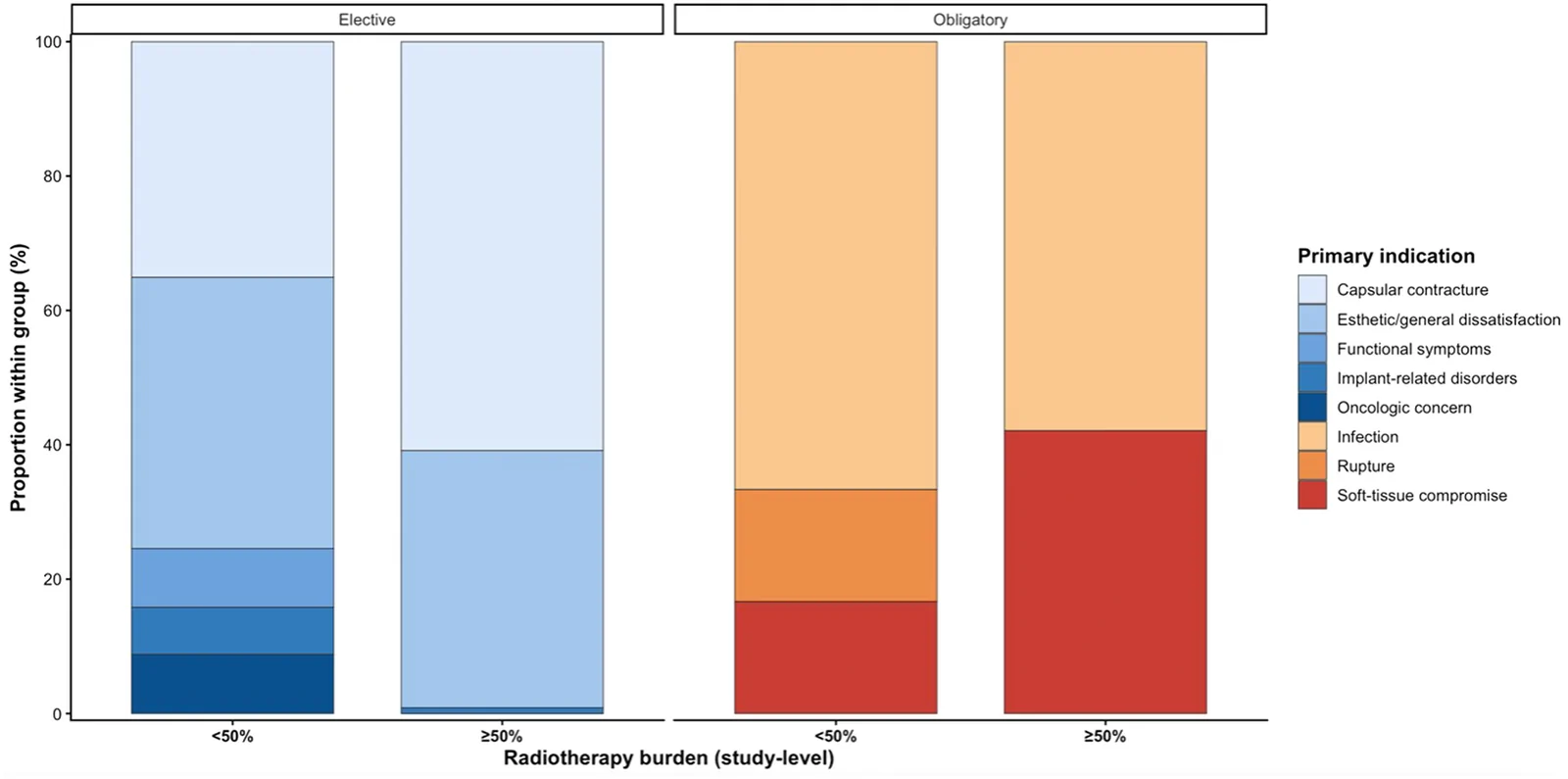
Exploratory study-level analysis of the distribution of primary elective and obligatory indications for autologous conversion, stratified by radiotherapy burden (RT <50%vs ≥50%), restricted to the six studies reporting a single main indication per patient.

Indications and motivations for implant removal leading to autologous conversion are summarized in Table 4 using complementary patient-level and event-level syntheses. In patient-level analyses restricted to exclusive reporting cohorts (Panel A; 6 studies; 473 patients), elective surgery was the most frequently reported primary indication for conversion (86.0%), while obligatory surgery accounted for 9.5%, and the primary indication was unknown in 4.4%. Among elective conversions, capsular contracture was the leading driver (57.2%), followed by aesthetic or general dissatisfaction (38.6%). Among obligatory conversions, infection and exposure-related complications predominated, with infection alone reported in 33.3%, soft-tissue compromise (exposure/extrusion/skin necrosis) in 20.0%, and a combined infection-or-exposure category in 44.4%. An exploratory descriptive stratification by study-level radiotherapy burden (<50% versus ≥50%) is presented in Fig. 2.

**Table 4:** Indications and motivations for implant removal leading to autologous conversion.

**Panel A. Patient-level primary indication (exclusive reporting cohorts) – Six studies** *(Each patient contributes to one category only; mutually exclusive reporting)*					
**Category**		**Contributing studies / total**	**Patients (n)**	**Total patients analysed**	**Overall proportion (%)**
**Elective Surgery**		6 / 6	407	473	86.0%
	Capsular contracture	5 / 6	233	407	57.2%
	Aesthetic or general dissatisfaction	5 / 6	157	407	38.6%
	Implant-related disorders*	2 / 6	7	407	1.7%
	Functional symptoms (pain / discomfort)	1 / 6	5	407	1.2%
	Oncologic concern §	1 / 6	5	407	1.2%
**Obligatory Surgery**		6 / 6	45	473	9.5%
	Infection	3 / 6	15	45	33.3%
	Rupture	1 / 6	1	45	2.2%
	Soft-tissue compromise††	2/6	9	45	20%
	Infection or Exposure‡	2 / 6	20	45	44.4%
**Unknown**		6 / 6	21	473	4.4%
**Panel B. Event-level reasons (non-exclusive reporting cohorts) – Twelve studies** *(Multiple indications per patient possible; non-mutually exclusive reporting)*					
**Category**		**Contributing studies / total**	**Events (n)**	**Total events analysed**	**Overall proportion (%)**
**Elective Surgery**		12 / 12	1172	1419	82.5%
	Capsular contracture	11 / 12	549	1172	46.8%
	Aesthetic or general dissatisfaction	6 / 12	278	1172	23.7%
	Functional symptoms (pain/discomfort)	7 / 12	170	1172	14.5%
	Implant-related disorders*	6 / 12	127	1172	10.8%
	Skin changes†	4 / 12	36	1172	3.0%
	Oncologic concern §	2 / 12	12	1172	1.0%
**Obligatory Surgery**		12 / 12	247	1419	17.4%
	Infection	9 / 12	91	247	36.8%
	Rupture	8 / 12	93	247	37.6%
	Soft-tissue compromise††	5 / 12	63	247	25.5%

⁎ Implant-related disorders include asymmetry, malposition, rippling, animation deformity, or “frozen breast”. † Skin changes without necrosis. ‡ « Infection or exposure » refers to studies reporting a combined category not separable into infection vs exposure/extrusion. § Oncological concern refers to locoregional recurrence cancer or a metachronous breast cancer. †† Soft-tissue compromise refers to implant exposure, extrusion, or skin necrosis.

In event-level analyses from non-exclusive reporting cohorts (Panel B; 12 studies; 1419 total events), elective reasons represented 82.5% of reported events, while obligatory indications accounted for 17.4%. Within elective events, capsular contracture remained the most frequently reported event (46.8%), followed by aesthetic or general dissatisfaction (23.7%), functional symptoms such as pain or discomfort (14.5%), and implant-related disorders accounted for 10.8% of events. Within obligatory events, rupture accounted for 37.6%, infection for 36.8%, and soft-tissue compromise for 25.5%.

### Safety outcomes

**Fig. 3:**
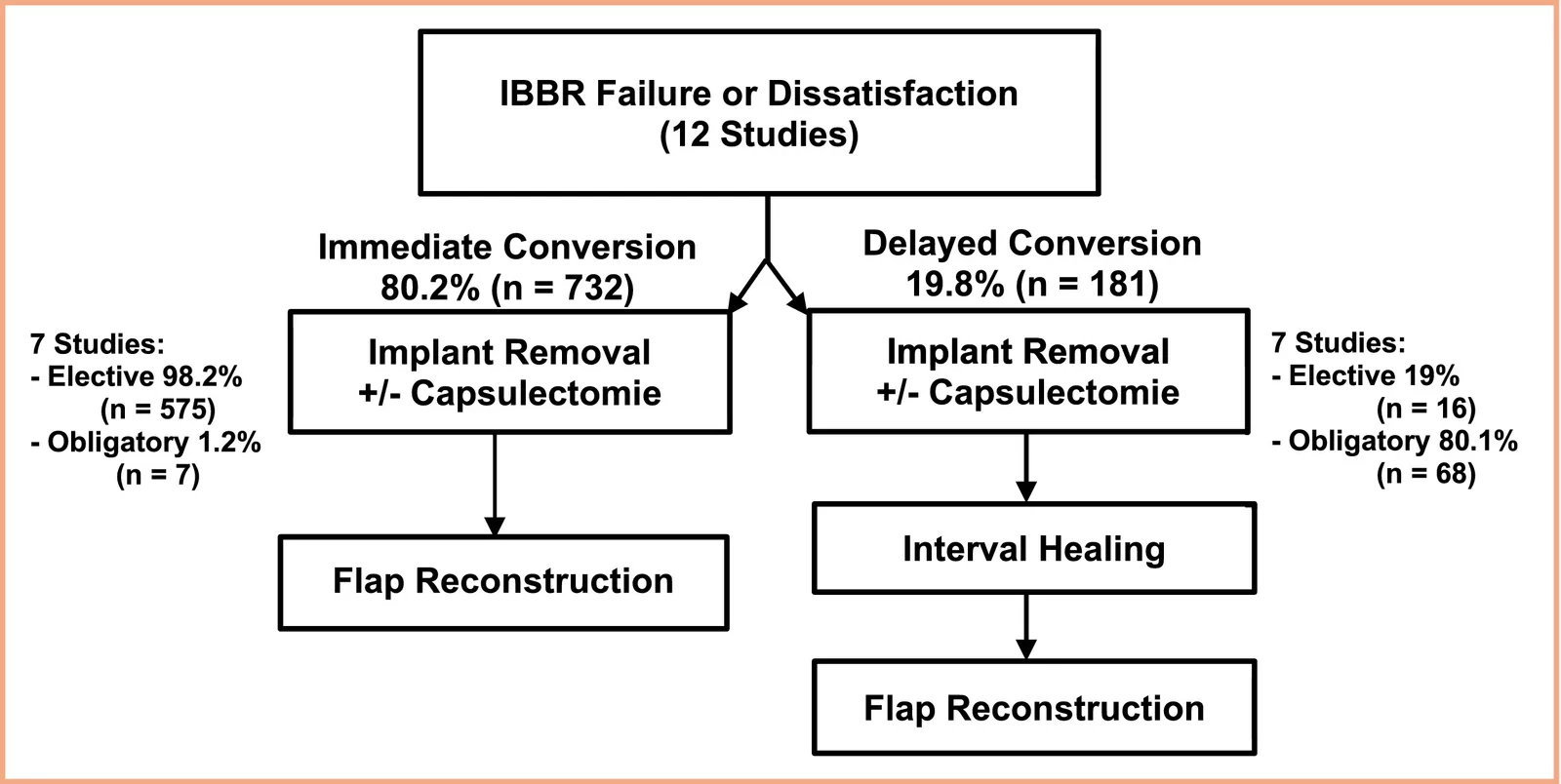
Timing and surgical pathways to autologous conversion following IBBR failure or dissatisfaction, among 12 reporting studies.

Across the included studies, 12 provided extractable data to characterize pre-conversion timing pathways. Autologous conversion after IBBR failure or dissatisfaction was most commonly performed as a single-stage immediate conversion (explantation ± capsulectomy with flap reconstruction during the same operation), whereas a two-stage delayed conversion (implant removal ± capsulectomy followed by an interval of healing before flap reconstruction) was less frequently reported. Among these, seven studies reported sufficient data to determine the distribution of elective versus obligatory indications within each pathway. Overall, approximately 80% of conversions followed a single-stage strategy, whereas 20% were managed using a staged approach. The staged pathway primarily reflected obligatory implant removal (e.g., infection or exposure) requiring explantation without immediate reconstruction, whereas elective conversions were predominantly performed as single-stage immediate conversions (Fig. 3).

**Fig. 4:**
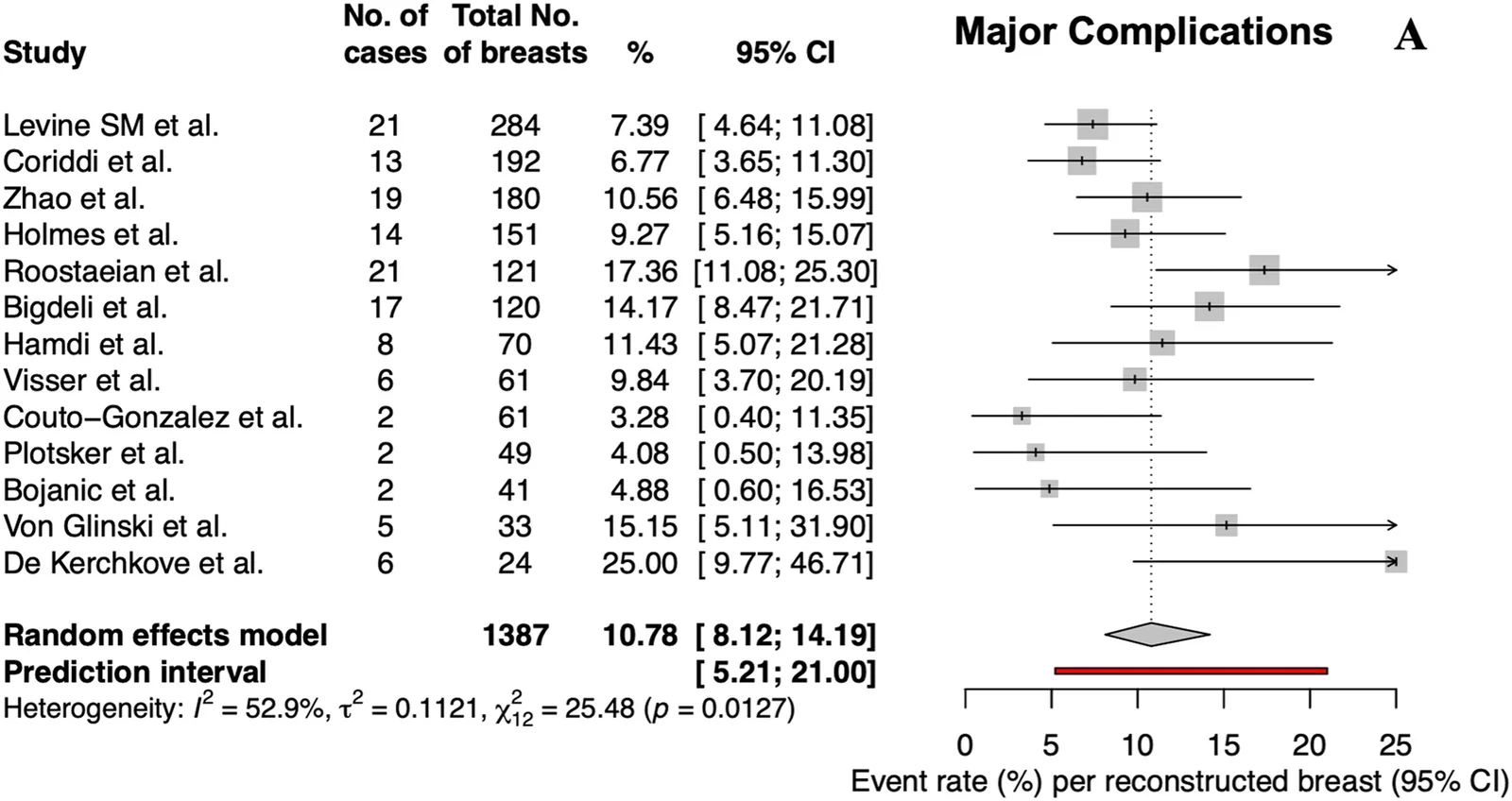

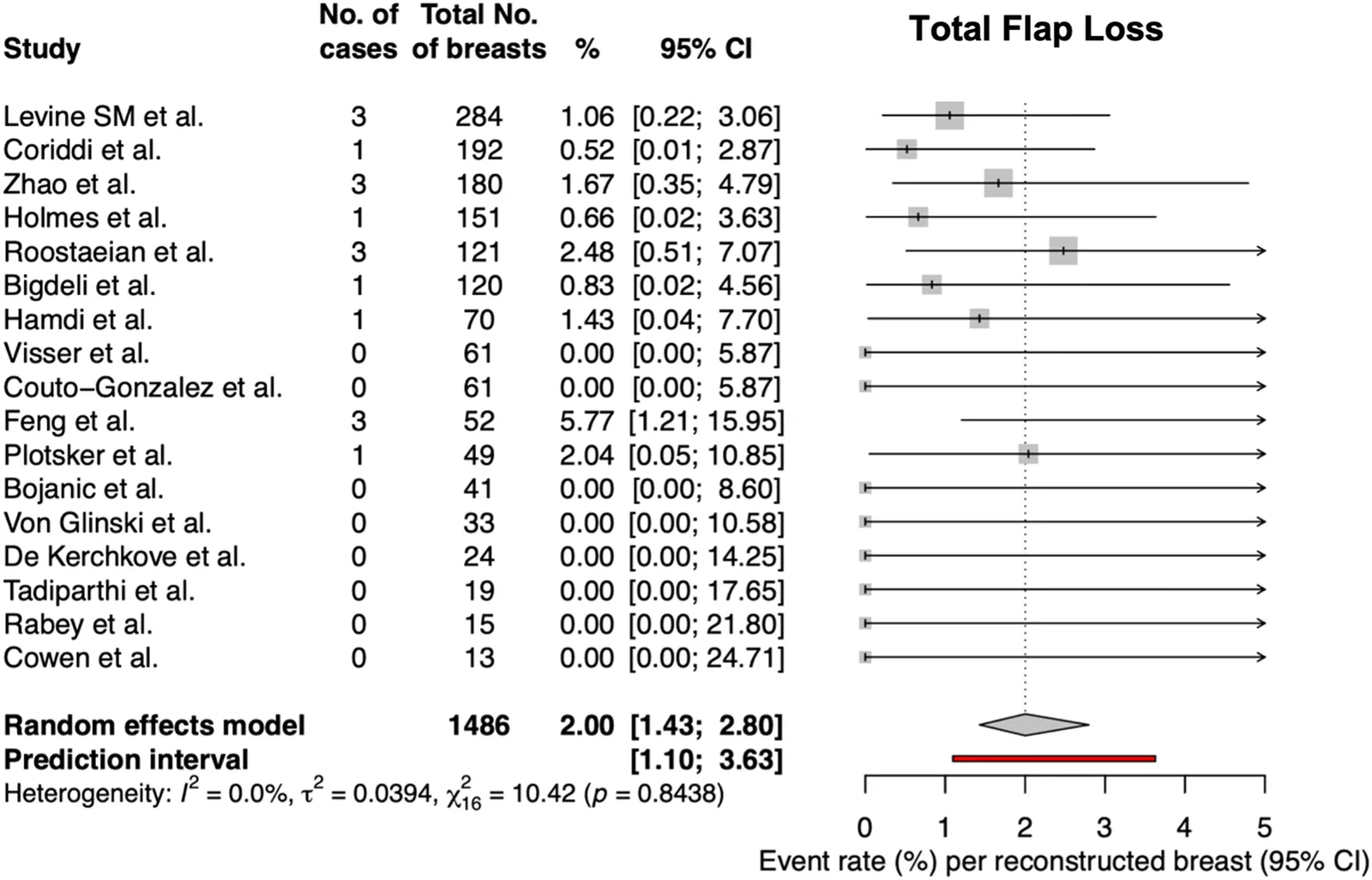
Pooled event rates of major complications (A) and total flap loss (B) after autologous conversion (per reconstructed breast).

In per-breast single-arm pooling, the pooled major complications rate after autologous conversion was 10.8% (95% CI [8.12–14.19]) across 1387 reconstructed breasts, with moderate heterogeneity (I² = 52.9%) and a prediction interval of [5.21–21.0]% (Fig. 4*A*). Six studies were excluded from this meta-analysis because major complications could not be reliably extracted or mapped to return-to-operating-room events based on the available reporting. Major events were most commonly related to flap-specific complications, including vascular compromise requiring anastomotic revision, reoperation for hematoma, or flap loss. The pooled total flap loss rate was 2.00% (95% CI [1.43–2.83]; I² = 0.0%) across 1486 converted breasts, with a prediction interval of [1.10–3.63]% (Fig. 4*B*). Two studies were excluded from this meta-analysis because total flap loss was not explicitly reported. Sensitivity analyses excluding studies reporting pedicled flaps did not materially alter these estimates.

**Fig. 5:**
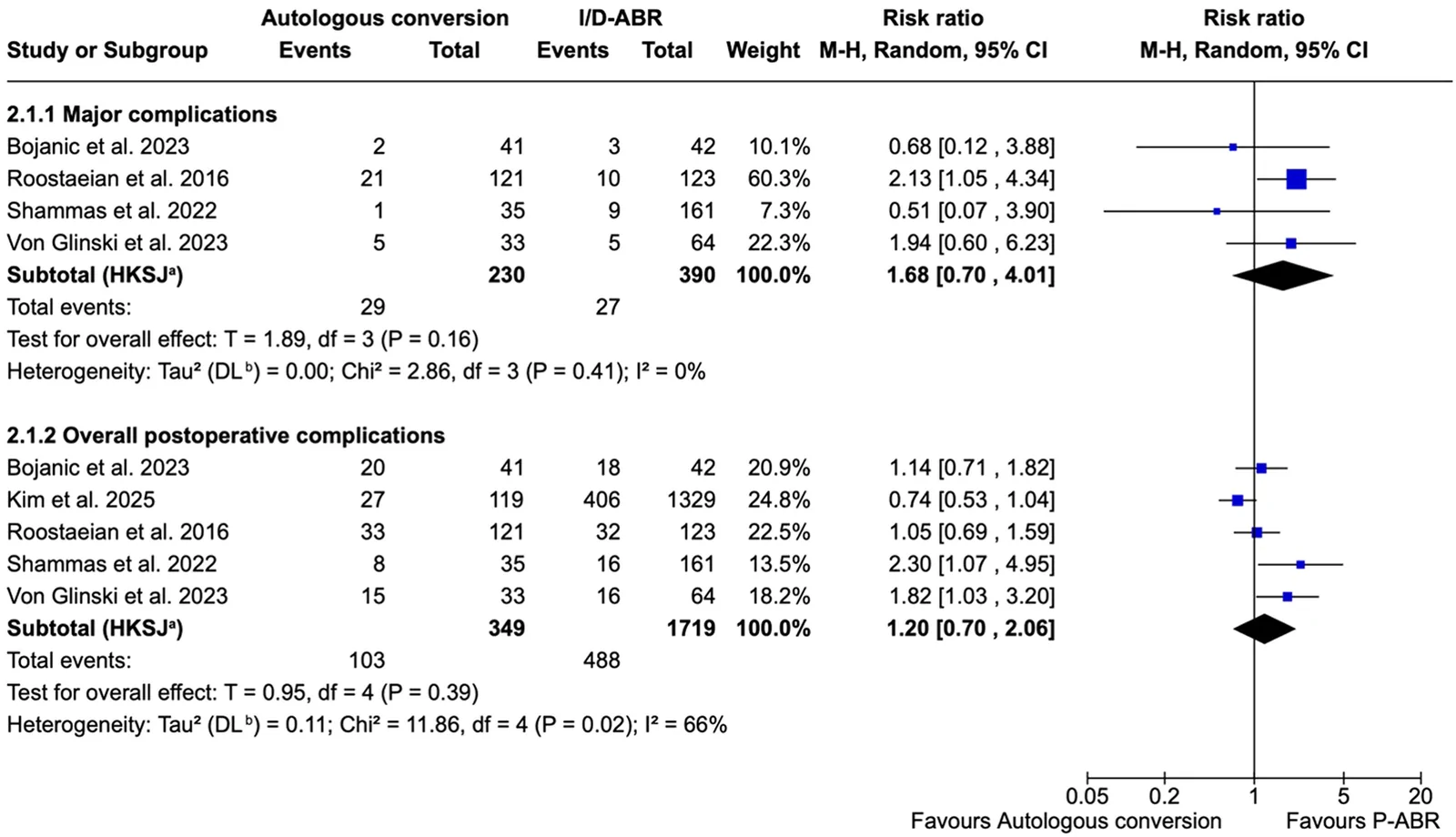
Comparative meta-analysis of major and overall postoperative complications after autologous conversion versus P-ABR.

In comparative analyses, no statistically significant difference in major complications was identified between autologous conversion and P-ABR (RR 1.68; 95% CI [0.70–4.01]; I² = 0%) (Fig. 5). Overall postoperative complications were also not statistically different (RR 1.20; 95% CI [0.70–2.06]), with substantial heterogeneity (I² = 66%). Given the limited number of available studies and the small number of reported total flap loss events, no pooled comparative analysis of total flap loss could be performed, and risk-adjusted or subgroup analyses were not feasible.

### Aesthetic outcomes

Revision procedures for aesthetic refinement were inconsistently reported. Among nine studies, 41.3% of patients (311/752) underwent at least one secondary procedure, most frequently fat grafting and contralateral symmetrisation.

Aesthetic outcomes were reported in nine studies using three distinct assessment approaches (Table 5): blinded surgeon panel photographic evaluation using a 5-point Likert scale (2 studies), patient-reported outcomes using the BREAST-Q© “Satisfaction with Breasts” domain (6 studies), and non-validated patient questionnaires assessing perceived aesthetic improvement (2 studies). Study designs included comparative cohorts versus P-ABR or continued IBBR, as well as within-cohort pre-post analyses after conversion.

**Table 5:** Aesthetic outcomes after autologous conversion following IBBR.

Study	Study comparator	Groups (n)	Aesthetic assessor	Assessment method	Timing of assessment (months)	Main aesthetic finding
Visser et al. 2010^57^	Pre-post Conversion	Pre: 29	Blinded surgeon panel (n = 5)	Standardized photographic assessment ; 5-point Likert scale	≥12	Improved vs baseline (volume; shape; symmetry; all p < 0.001)
		Post: 31				
Von Glinski et al. 2023^14^	Conversion vs P-ABR	Conversion: 23	Blinded surgeon panel (n = 5)	Standardized photographic assessment ; 5-point Likert scale	≥12 (both groups)	Lower vs P-ABR across multiple domains (all p ≤ 0.01)
		P-ABR: 47				
Feng et al. 1994^56^	Conversion only	Conversion: 25	Patient-reported	Ad hoc questionnaire	≥3	Patient-reported aesthetic improvement
De Kerchkove et al. 2023^64^	Conversion only	Conversion: 21	Patient-reported	Ad hoc questionnaire	≥12	Patient-reported aesthetic improvement
Tadiparthi et al. 2013^59^	Conversion vs IBBR	Conversion: 18	Patient-reported	BREAST-Q© (Satisfaction with Breasts)	≥12	Higher vs IBBR (p = 0.005)
		IBBR: 52				
Coriddi et al. 2019^21^	Pre-post Conversion	Pre: 23	Patient-reported	BREAST-Q© (Satisfaction with Breasts)	15 (range 1.5–36)	Improved vs baseline (p < 0.001)
		Post: 23				
Cowen et al. 2020^62^	Conversion vs IBBR	Conversion: 20	Patient-reported	BREAST-Q© (Satisfaction with Breasts)	≥12 (both groups)	Higher vs IBBR (p < 0.0001)
		IBBR: 32				
Shammas et al. 2022^37^	Conversion vs P-ABR	Conversion: 23	Patient-reported	BREAST-Q© (Satisfaction with Breasts)	≥12 (both groups)	Lower vs P-ABR (p = 0.04)
		P-ABR: 94				
Plotsker et al. 2024^66^	Pre-post Conversion	Pre: 18	Patient-reported	BREAST-Q© (Satisfaction with Breasts)	≥12	Improved vs baseline (p < 0.001)
		Post: 17				
Kim et al. 2025^67^	Conversion vs P-ABR	Conversion: 41	Patient-reported	BREAST-Q© (Satisfaction with Breasts)	≥3 (both groups)	No difference vs P-ABR (p = 0.161)
		P-ABR: 480				

P-ABR: primary autologous breast reconstruction; IBBR: implant-based breast reconstruction.

Two studies evaluated aesthetic outcomes based on standardized photographs rated by a blinded panel of five surgeons using a 5-point Likert scale. In a pre-post analysis Visser et al. reported significantly favorable aesthetic outcomes after conversion, with improvements observed for breast volume, shape, and symmetry. In contrast, Von Glinski et al., in a comparative cohort design with evaluation performed at ≥12 months in both groups, reported significantly lower surgeon-rated aesthetic scores in the conversion group compared with P-ABR across multiple domains, including breast shape, volume, symmetry, nipple-areolar complex position, inframammary fold position, and overall breast appearance.

Patient-reported breast satisfaction assessed by BREAST-Q© “Satisfaction with Breasts” was available in six studies. Pre-post analyses consistently reported significant improvement after conversion. In comparative cohorts, BREAST-Q© scores were higher in conversion cohorts than in IBBR cohorts, while comparisons with P-ABR were variable across studies. Two additional single-arm studies using non-validated questionnaires reported that most patients perceived an aesthetic improvement following conversion.

## Discussion

Breast reconstruction should be considered a longitudinal reconstructive pathway rather than a single surgical event. Outcomes evolve over time under the influence of oncologic therapies, implant-related morbidity, and cumulative revisions.^26^ Within this trajectory, implant failure or persistent dissatisfaction represents a critical decision point that may prompt transition to autologous reconstruction.^21^

### Clinical profile and oncologic exposures

The baseline clinical profile of patients undergoing conversion appeared broadly comparable to that reported in contemporary reconstructive cohorts. Study-level age, body mass index, comorbidity burden, and chemotherapy exposure were within the ranges described in large IBBR and ABR populations.^27^, ^28^, ^29^, ^30^, ^31^, ^32^, ^33^

Radiation therapy exposure, in contrast, was reported in approximately 58% of converted patients. Although radiotherapy rates vary substantially according to tumor stage and oncologic risk profile, this proportion appears higher than that typically observed in unselected mastectomy reconstruction populations.^27^^,^^33^, ^34^, ^35^, ^36^ These findings suggest that radiation-associated soft-tissue injury likely contributes to implant-related morbidity and subsequent conversion. Radiation, however, does not appear to be the sole determinant.

These observations support a nuanced interpretation. While radiation therapy may increase vulnerability to implant-related complications, conversion patients were not otherwise identifiable as a distinctly high-risk subgroup at baseline. Rather, IBBR appears to expose a broad spectrum of patients to progressive long-term morbidity. Dissatisfaction and complications may accumulate over years before conversion is pursued. Consistent with this interpretation, time to conversion was frequently prolonged, and conversion was not uniformly preceded by multiple prosthetic revision attempts. Conversion should therefore not be viewed solely as a last-resort salvage after repeated implant failure. Instead, it may represent an earlier strategic transition within the reconstructive pathway, particularly given prior evidence suggesting diminishing returns with iterative implant revisions.^23^^,^^37^

### Indications and motivations leading to autologous conversion

Across studies, conversion was predominantly elective and driven by chronic implant-related morbidity, particularly capsular contracture and persistent dissatisfaction, often accompanied by pain or functional discomfort. Obligatory explantation for acute implant-threatening complications such as infection or exposure was less frequent.

The elective-obligatory distinction has practical implications. These scenarios differ in timing, tissue quality, urgency, patient expectations, and operative strategy.^38^ Elective conversions were more frequently performed as single-stage procedures. Obligatory indications more often required staged management, with initial explantation followed by delayed flap reconstruction after interval healing. In our synthesis, delayed conversions were uncommon overall but disproportionately associated with obligatory indications, consistent with a risk-mitigation strategy in infected or compromised tissues.

Beyond failure management, autologous conversion provides a biologically coherent reconstructive solution. Replacement of compromised IBBR with well-vascularized autologous tissue may restore soft-tissue quality, improve long-term stability across weight and age variation, enhance patient-reported satisfaction, and potentially improve sensory recovery when combined with nerve coaptation techniques.^17^^,^^39^^,^^40^

Our exploratory analysis further suggested that higher radiotherapy exposure may shift conversion drivers toward capsular pathology and soft-tissue compromise. This pattern is biologically plausible given radiation-associated fibrosis, progressive soft-tissue deterioration, and microvascular and stromal changes that may impair tissue resilience.^41^^,^^42^ These mechanisms likely contribute to the predominance of capsular contracture among elective conversions and exposure-related complications among obligatory cases in irradiated cohorts.^43^ Although post-mastectomy radiation therapy is consistently associated with higher risks of complications and reconstructive failure in IBBR, rising from approximately 3–5% implant failure to beyond 10% in studies,^8^, ^9^, ^10^^,^^44^^,^^45^ ABR may demonstrate greater resilience in this setting through the introduction of well-vascularized tissue and improved soft-tissue quality.^46^, ^47^, ^48^, ^49^

### Surgical safety of autologous conversion

From a surgical safety perspective, this synthesis provides reassuring evidence. Pooled single-arm analyses demonstrated an approximately 11% major complication rate, with moderate heterogeneity. Total flap loss remained low at 2%, without observed heterogeneity. These estimates appear broadly consistent with reported benchmarks for ABR more generally, suggesting that autologous conversion can be performed without a clear excess risk of flap loss or major complications despite prior implant surgery, scarring, and frequent oncologic treatment exposure.^50^, ^51^, ^52^, ^53^

However, these pooled estimates were not risk-adjusted for factors that likely influence outcomes, including indication for explantation, degree of soft-tissue compromise, and center-level technical practices such as extent of capsulectomy. Indeed, outcomes likely differ between elective and obligatory conversion pathways. Comparative evidence remains inconsistent: Bigdeli et al. reported higher major complications rate in obligatory compared with elective conversions, whereas Zhao et al. did not observe a statistically significant difference between these subgroups.^38^^,^^54^ Operative timing further complicates interpretation. Conversion may be performed immediately after implant removal or in a staged manner. Delayed approaches were disproportionately associated with obligatory indications, where unplanned implant removal for acute complication, is follow by interval healing who could mitigate risk in compromised fields.

Comparative analyses reinforced these findings and did not demonstrate statistically significant differences in major or overall complications relative to P-ABR. However, these findings must be interpreted cautiously given the wide confidence intervals, observational design and potential confounding by indication.

Overall, the cumulative signal supports that autologous conversion can be performed with acceptable safety, even in patients with prior infection, chronic implant inflammation, or radiation exposure.

### Aesthetic outcomes

Aesthetic outcomes were less consistently reported but remains central to the value proposition of conversion. Approximately 41% of patients underwent secondary refinement procedures, most commonly fat grafting or contralateral symmetrization. This highlights that conversion, similar to P-ABR, often represents a transition rather than a definitive endpoint. Patients should be counseled preoperatively that additional staged refinements may be required to optimize contour, projection and symmetry.

Despite heterogeneity in assessment tools and study design, all pre-post analyses demonstrated improvement in aesthetic outcomes and breast satisfaction following conversion. Comparative cohorts generally reported higher satisfaction versus continued IBBR pathway, consistent with prior evidence.^55^ Comparisons with P-ABR were inconsistent, with some studies reporting lower surgeon-rated scores and others demonstrating no difference.

Taken together, available evidence suggests that conversion is associated with meaningful improvement from the pre-conversion baseline or continued IBBR pathway. A residual difference relative to P-ABR may persist in some settings, potentially reflecting cumulative tissue injury, scarring and envelope constraints at the time of conversion.

### Limitations

Several important limitations inherent to the current literature should be acknowledged. The available evidence is dominated by retrospective observational cohorts, with 16 of 19 studies using a retrospective design and most reporting single-arm cohorts. Outcome measures and definitions were rarely standardized and varied substantially across studies, particularly regarding elective versus obligatory indications, complication grading, secondary refinement procedures, aesthetic assessment methods and timing of outcome evaluation, which ranged from a few months to several years. This heterogeneity may have introduced bias in outcome interpretation and increased the risk of misclassification and underreporting, especially for minor complications. Indications or motivations were frequently reported as non-mutually exclusive events, precluding formal pooling and limiting patient-level interpretation. Comparative analyses remain susceptible to confounding by indication, as patients undergoing conversion may follow a more complex reconstructive trajectory than those selected for P-ABR or continued IBBR. Moreover, 10 of 19 studies did not include a control group, restricting the extent of comparative analysis possible from the available data. Publication bias was not formally assessed and cannot be excluded. The included studies spanned a broad period from 1994 to 2025, during which implant technology, radiation protocols, microsurgical techniques, and perioperative care evolved substantially. However, most evidence was relatively contemporary, with 13 of 19 studies published within the last decade, five between 2010 and 2013, and only one before 2000, which may partially mitigate (but not eliminate) the limitations related to temporal heterogeneity. Finally, most data originated from tertiary referral centers, which may limit generalizability.

### Perspectives

**Fig. 6:**
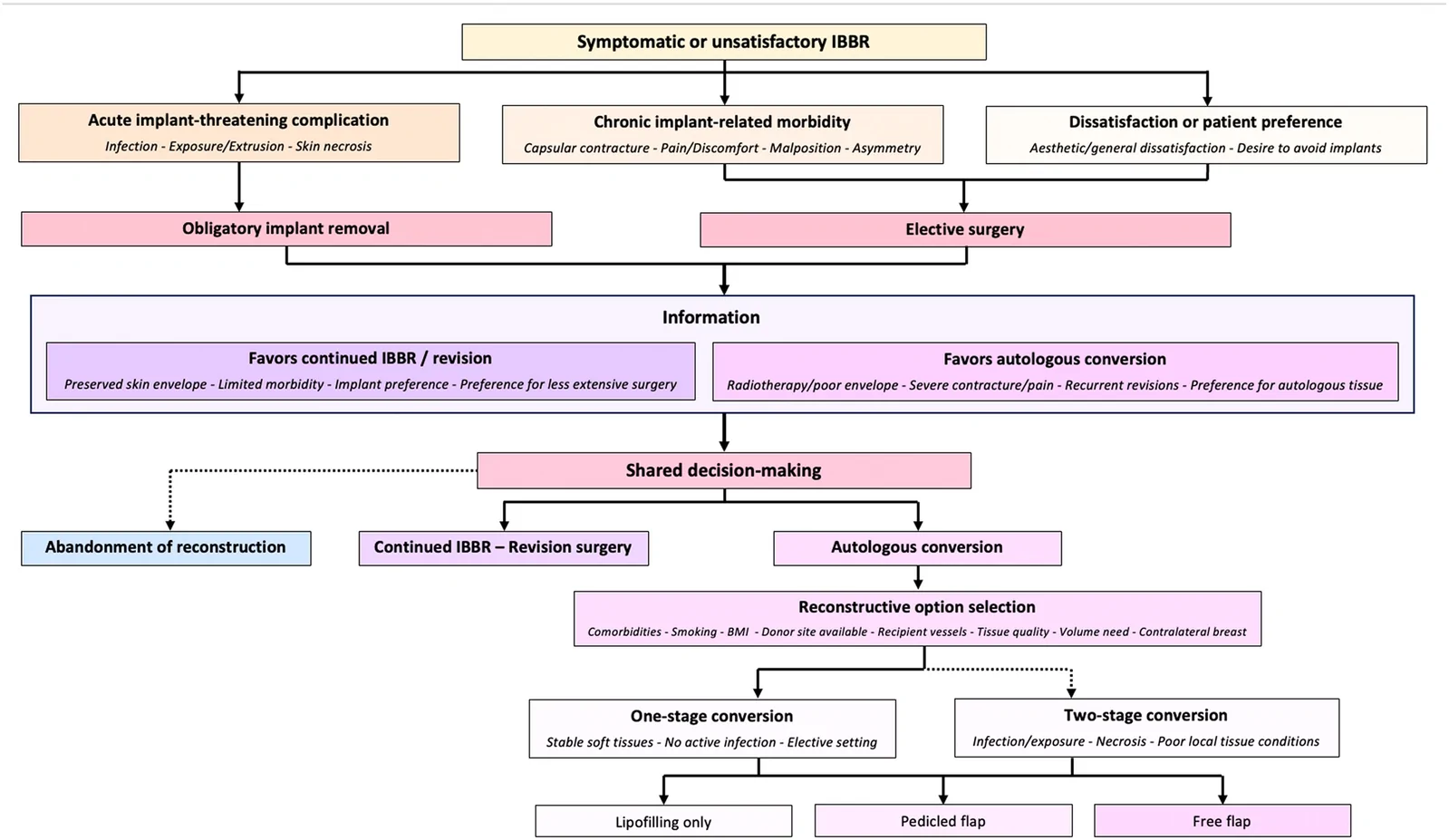
Proposed decision-making algorithm for symptomatic or unsatisfactory implant-based breast reconstruction (IBBR).

Based on the present synthesis, we propose a practical decision-making algorithm for patients presenting with symptomatic or unsatisfactory IBBR, integrating the potential role of autologous conversion (Fig. 6). This algorithm is intended as a shared decision-making aid to help surgeons and patients evaluate the available options according to patient-related factors, local tissue conditions, and individual preferences. At each stage of the reconstructive course, patients should be informed of the benefits and limitations of each strategy, so that the selected approach is not only surgically feasible, but also durable and aligned with patient expectations. Future high-quality, ideally prospective and comparative studies with appropriate control groups are needed to refine this decision-making approach and identify which strategy provides the best balance between safety, aesthetic outcome, durability, and patient-centered satisfaction.

## Conclusion

Autologous conversion after IBBR is most commonly performed electively for chronic implant-related morbidity, particularly capsular contracture and persistent dissatisfaction, while a smaller but distinct subgroup undergoes obligatory explantation for acute implant-threatening complications. Across heterogeneous observational cohorts, flap reliability appears preserved, with low pooled total flap loss and acceptable major or overall complication rates despite prior surgery and frequent radiation exposure. Available evidence suggests that autologous conversion is associated with improvement in aesthetic outcomes compared with the pre-conversion state or continued IBBR pathway. Taken together, these findings support autologous conversion as a clinically meaningful and reliable reconstructive strategy when IBBR becomes symptomatic, fails, or remains unsatisfactory.

## Funding

None.

## Conflicts of interest

None declared.

## Ethical approval

Not required.
